# Differential Phosphorylation of RNA Polymerase III and the Initiation Factor TFIIIB in *Saccharomyces cerevisiae*

**DOI:** 10.1371/journal.pone.0127225

**Published:** 2015-05-13

**Authors:** Jaehoon Lee, Robyn D. Moir, Ian M. Willis

**Affiliations:** 1 Department of Biochemistry, Albert Einstein College of Medicine, Bronx, New York, United States of America; 2 Department of Systems and Computational Biology, Albert Einstein College of Medicine, Bronx, New York, United States of America; University of Padova, ITALY

## Abstract

The production of ribosomes and tRNAs for protein synthesis has a high energetic cost and is under tight transcriptional control to ensure that the level of RNA synthesis is balanced with nutrient availability and the prevailing environmental conditions. In the RNA polymerase (pol) III system in yeast, nutrients and stress affect transcription through a bifurcated signaling pathway in which protein kinase A (PKA) and TORC1 activity directly or indirectly, through downstream kinases, alter the phosphorylation state and function of the Maf1 repressor and Rpc53, a TFIIF-like subunit of the polymerase. However, numerous lines of evidence suggest greater complexity in the regulatory network including the phosphoregulation of other pol III components. To address this issue, we systematically examined all 17 subunits of pol III along with the three subunits of the initiation factor TFIIIB for evidence of differential phosphorylation in response to inhibition of TORC1. A relatively high stoichiometry of phosphorylation was observed for several of these proteins and the Rpc82 subunit of the polymerase and the Bdp1 subunit of TFIIIB were found to be differentially phosphorylated. Bdp1 is phosphorylated on four major sites during exponential growth and the protein is variably dephosphorylated under conditions that inhibit tRNA gene transcription. PKA, the TORC1-regulated kinase Sch9 and protein kinase CK2 are all implicated in the phosphorylation of Bdp1. Alanine substitutions at the four phosphosites cause hyper-repression of transcription indicating that phosphorylation of Bdp1 opposes Maf1-mediated repression. The new findings suggest an integrated regulatory model for signaling events controlling pol III transcription.

## Introduction

RNA polymerase (pol) III synthesizes highly abundant tRNAs and 5S RNA and numerous less abundant small non-translated RNAs. The high energetic cost of tRNA and 5S RNA synthesis requires tight regulation of transcription to achieve metabolic economy. In yeast and higher eukaryotes, this metabolic economy is enforced by Maf1, a nutrient- and stress-regulated repressor of pol III transcription [1, 2]. Transcriptional repression by Maf1 is negatively regulated by phosphorylation. Yeast Maf1 is phosphorylated by PKA, Sch9, and TORC1 at multiple sites during exponential growth. These modifications promote its localization in the cytoplasm and/or inhibit its interaction with the polymerase in the nucleus. Under conditions that restrict pol III transcription, the activity of the aforementioned pro-growth kinases is diminished, Maf1 is dephosphorylated and the protein accumulates in the nucleus where it can bind to its targets, Brf1 and the largest subunit of pol III, Rpc160, to repress transcription (reviewed in [3]).

Pol III transcription in cells growing in nutrient-replete conditions is not significantly decreased by simple nuclear accumulation of Maf1 or by preventing Maf1 phosphorylation [4–6]. Additionally, in vitro studies show that pol III bound to preinitiation complexes or in elongation complexes is protected from repression by recombinant Maf1 as is transcription initiation by pol III molecules undergoing facilitated recycling [7, 8]. These observations suggest that Maf1 inhibits transcription only after the polymerase has dissociated from the DNA. How this is achieved is not well understood. However, facilitated recycling requires a dissociable three subunit complex of the polymerase comprising the TFIIF-like subunits, C53 and C37, along with C11 [9] and C53 is phosphoregulated during Maf1-dependent repression [10]. Under stress conditions, the Cdc-like kinase Kns1 phosphorylates C53 at a single site thereby priming the protein for phosphorylation by a specific GSK-3 family kinase, Mck1 [10]. Importantly, alanine substitutions at the Kns1 and Mck1 sites in C53 limit the extent of repression but only in a sensitized strain that is compromised for facilitated recycling by a hypomorphic C11 protein [10, 11]. These findings, together with other observations described below, suggest that additional signaling events contribute to the regulation of pol III transcription, both to enable Maf1-dependent repression in response to stress and to oppose Maf1 inhibition under optimal growth conditions.

The initiation factor TFIIIB (comprised of the TFIIB-related factor Brf1, TATA-box binding protein TBP and the SANT domain-containing Bdp1 protein) is well established as a regulatory target in higher eukaryotes. TFIIIB subunit abundance and interactions are regulated by oncogenic proteins and tumor suppressors (reviewed in [12, 13]) and subunit gene expression is also controlled by the activity of Erk and JNK kinases [14–16]. TFIIIB function is regulated by CK2 which associates with pol III-transcribed genes and activates pol III transcription [17, 18]. Both Brf1 and Bdp1 are phosphoproteins [19–21] and their regulated phosphorylation during the cell-cycle by CK2 [22] and Plk1 kinases [23] correlates with transcription. In yeast, CK2 is required for robust pol III transcription in vitro [24] and is associated with tRNA genes in both *S*. *cerevisiae* and *S*. *pombe* [25–27]. Although decreased pol III transcription caused by DNA damage correlates with reduced CK2 activity associated with TBP [25] and growth to stationary phase results in a decrease in Brf1 protein abundance [28], little has been reported on the regulation of TFIIIB components in this system.

Yeast phospho-proteomic studies have identified a subset of pol III subunits and all TFIIIB subunits as phosphoproteins [29, 30]. We systematically examined all 17 subunits of pol III along with the three subunits of TFIIIB for evidence of differential phosphorylation in response to inhibition of TORC1. A relatively high stoichiometry of phosphorylation was observed for several of these proteins and the Rpc82 subunit of pol III and the Bdp1 subunit of TFIIIB were found to be differentially phosphorylated. Bdp1 is phosphorylated on four major sites in logarithmically growing cells and is variably dephosphorylated under conditions that inhibit pol III transcription. Consistent with the kinase consensus motifs at these positions, Bdp1 is phosphorylated by PKA, Sch9 and CK2 kinases in vitro. A Bdp1 4SA mutant allows hyper-repression of transcription indicating that phosphorylation of Bdp1 at these sites opposes Maf1-mediated repression.

## Materials and Methods

### Yeast stains and northern analysis

Tagged yeast strains were generated by standard PCR-based methodology [31]. Strains (S1 Table) were derived from W303 or S288C and grown in YPD or synthetic complete medium as required with or without drug treatment. Doubling times of yeast strains were determined by real time monitoring in a Bioscreen C instrument and represent the average of technical triplicates and biological duplicates for each strain [32]. Cultures for RNA analysis were grown to OD_600_ 0.4–0.5 and treated with drug vehicle (DMSO), rapamycin (0.2 μg/mL), chlorpromazine (250 mM) or methyl methanesulfonate (0.08% w/v) for one hour, or with tunicamycin (2.5 μg/mL, 3 hours). RNA extraction, Northern blot analysis, and quantitation were carried out as described [33]. Analog-sensitive strains were grown to mid-log phase and treated with 0.1 μM 1NM-PP1 or drug vehicle (DMSO) for 1 hour as described previously [34].

### Analysis of protein phosphorylation and immunoprecipitation

For analysis of phosphorylated species, lysates were prepared by TCA precipitation of 10 ml yeast culture at OD 1.0 (6% final TCA)[35]. Cells were harvested at room temperature, cell pellets were washed twice with 3M urea and extracts were prepared by glass bead breakage in 100 μl of urea lysis buffer (50 mM Tris [pH 7.5], 6 M urea, 1% SDS) containing 1X EDTA-free protease inhibitor cocktail (Roche). The lysates were separated on 25 μM Phos-tag acrylamide gels (Wako Chemicals) run at 120V for 2h and prepared for standard Western analysis as recommended [36]. Lambda phosphatase (NEB) treatment of extracts was performed in 1 mM MnCl_2_ at 30°C for 1hour prior to analysis on Phos-tag gels as above. For immunoprecipitation, extracts were prepared by glass bead breakage in 0.5 ml KBC100 breaking buffer containing protease and phosphatase inhibitors [34]. Immunoprecipitation was carried out with HA monoclonal antibody (12CA5, Roche) and protein G-Sepharose beads. Maf1 or Maf1myc was detected with a purified polyclonal Maf1 antibody [37] or anti-Myc monoclonal antibody (Roche). Individual lines, one lane wide, were analyzed using Peak Finder (Image Quant 5.0 software) to calculate peak areas after background subtraction. Peak Fitting software (Origin-Pro 8.1) was used to generate the phosphorylation patterns of proteins separated by Phos-tag acrylamide electrophoresis. Curve parameters were determined for untreated wildtype proteins (and dephosphorylated proteins where appropriate) and were used to constrain peak positions in the treated or mutant protein samples.

### Generation of chromosomal *BDP1* mutant alleles

Site-directed mutagenesis (QuikChange II, Stratagene) of a plasmid template pRS316 BDP1-3HA was used to generate *BDP1* alleles containing serine to alanine or glutamic acid substitutions. All mutants were confirmed by sequencing. Overlap PCR was used to generate fusion products containing *BDP1* promoter, coding and HA sequences and the KanMX4 selection cassette [38]. The resulting PCR products were transformed into a W303 *bdp1*Δ::natR strain that contained pRS316 BDP1-3HA. The rescuing plasmid was evicted by growth on FOA (confirmed by the absence of growth on SC-Ura). The integrity and identity of the integrated *BDP1* alleles was confirmed by sequencing of PCR products amplified from genomic DNA.

### Kinase assays

Bacterially expressed Bdp1 WT, Bdp1 2SA (S164/178A) and Bdp1 4SA proteins with a C-terminal 6 His motif were prepared under native conditions [39]. In vitro kinase reactions contained recombinant Bdp1 protein (100 ng), ^32^P-γ-ATP and kinase preparations of either murine PKA catalytic subunit (100 U, NEB), human CK2 (0.54 U, NEB) or yeast GST-Sch9 (4ul, wild type or kinase dead, KD) [10].

## Results

### Signaling by PKA and/or Sch9 suggests pol III transcriptional targets in addition to Maf1

**Fig 1:**
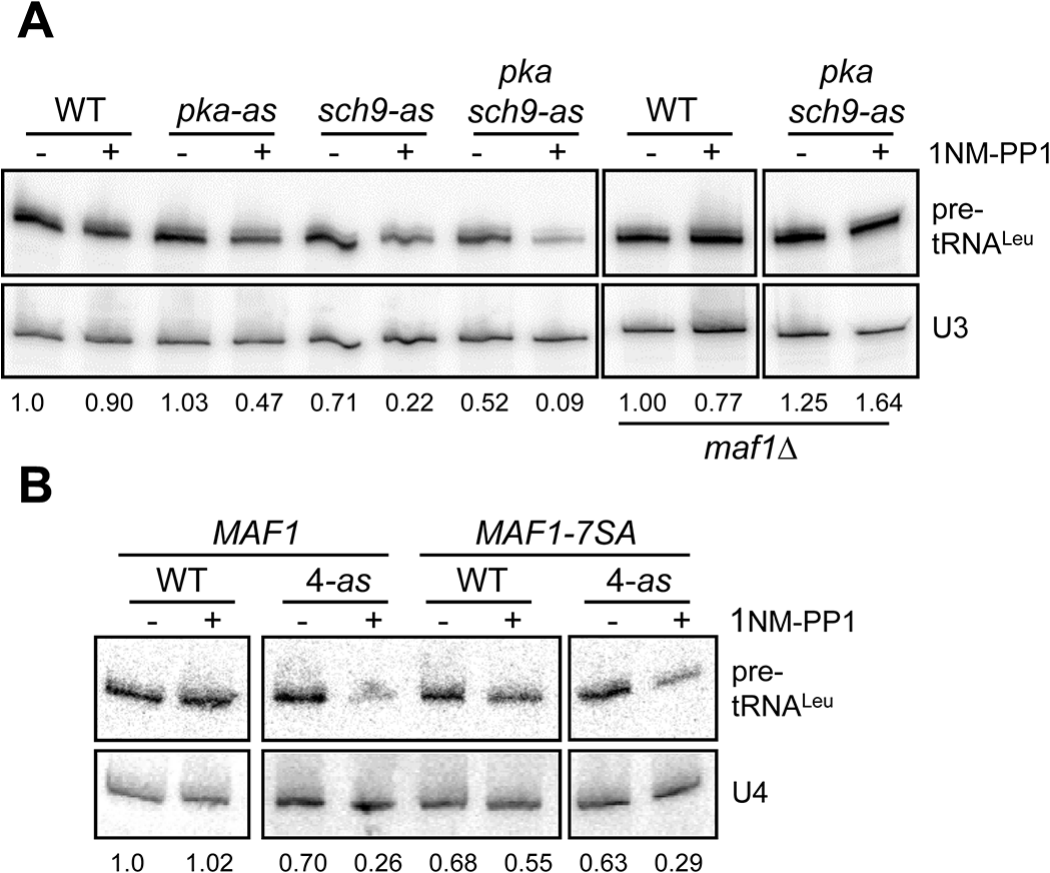
PKA and Sch9 regulate pol III transcription through additional effectors other than Maf1. Northern analysis of pol III transcription in analog-sensitive strains. *pka-as*, *sch9-as* and *pka-as sch9-as* strains (abbreviated to *4-as* in panel B) were treated with 1NM-PP1 or drug vehicle for 1 hour. **A.** Pol III transcription reported by the amount of pre-tRNA^Leu^ transcript is normalized to U3 snRNA, expressed relative to the untreated wild-type strain and indicated below each lane. Transcription is sensitive to loss of either PKA or Sch9 catalytic activity and the reduction is Maf1-dependent (right-most 2 lanes). Transcription for *maf1*Δ *pka-as sch9-as* normalized to its *maf1*Δ control. **B.** Pol III transcription is sensitive to loss of PKA and Sch9 activity in a strain that contains alanine-substituted Maf1 (*MAF1-7SA*). An ATP analog-insensitive *maf1*Δ::natR strain and a *pka-as sch9-as maf1*Δ::natR strain, each containing a plasmid with *MAF1* wild-type (WT) or 7SA alleles were assayed and quantified as in panel A using U4 snRNA for normalization. The framed regions in panels A and B represent non-adjacent lanes from the same gels.

We and others have shown that PKA and Sch9 redundantly phosphorylate yeast Maf1 at six sites (serines 90, 101, 177, 178, 209, 210) with a seventh site (serine 179) targeted specifically by Sch9 [5, 34, 35, 40–42]. Thus, the near complete loss of Maf1 phosphorylation at these sites in vivo under optimal growth conditions requires the simultaneous inhibition of both kinases. This was demonstrated by comparing the phosphorylation state of Maf1 in a series of ATP analog-sensitive strains (*pka-as*, *sch9-as and pka/sch9-as*) following the addition of 1NM-PP1 [34]. However, parallel experiments to examine the inhibitory effects of the ATP analog on pol III transcription in these strains have not previously been reported (see Fig 1). Interestingly, despite the ability of PKA and Sch9 to buffer one another with respect to Maf1 phosphorylation [34], 1NM-PP1 inhibition of either kinase caused a significant (>50%) reduction in pol III transcription as determined by pre-tRNA^Leu^ northern analysis (Fig 1A). In this assay, the level of short-lived pre-tRNA^Leu^ molecules reports global tRNA synthesis and therefore pol III transcription [10, 43]. Unlike the *pka-as* strain, the *sch9-as* strain showed a modest decrease in pre-tRNA^Leu^ synthesis in the absence of 1NM-PP1 with analog addition causing a substantial further decrease. This effect was amplified in the *pka/sch9-as* strain (Fig 1A). Notably, reduced transcription in either the absence or the presence of 1NM-PP1 was fully dependent on Maf1 since the changes were quantitatively blocked in the *pka/sch9-as maf1*∆ strain (Fig 1A, right hand lanes). In addition, we observed that the effect of 1NM-PP1 treatment on each of the three analog-sensitive strains (>50% reduction in pre-tRNA^Leu^ synthesis after 1 hour) was significantly greater than the effect of the Maf1 7SA mutant under optimal growth conditions (25% reduction or 0.75 ± 0.07, n = 8, SEM, relative to 1.0 for wild-type, in pre-tRNA^Leu^ synthesis with mutation of all seven PKA and Sch9 sites to alanine) [44]. This suggested that PKA and Sch9 may have other targets in the pol III machinery that impact the level of transcription. To test this possibility, we examined the effect of 1NM-PP1 on pre-tRNA^Leu^ synthesis in wild-type and *pka/sch9-as* strains in the presence or absence of Maf1 7SA. If control of pol III transcription by PKA and Sch9 is exercised exclusively through Maf1, 1NM-PP1 should have no effect in the *pka/sch9-as MAF1-7SA* strain. This is not the case (Fig 1B); pre-tRNA^Leu^ synthesis in the *pka/sch9-as MAF1-7SA* was significantly reduced (comparable to wild-type Maf1) upon addition of the analog. Thus, these data support the existence of one or more additional substrates for PKA and Sch9 in the pol III system and suggest that the putative substrates are phosphorylated under normal growth conditions and that their dephosphorylation promotes Maf1-dependent repression.

### Systematic analysis of phosphoregulation among pol III and TFIIIB subunits

To identify new phosphoregulated components of the pol III machinery, we used Phos-tag acrylamide gels and western analysis to systematically assess whether the 17 subunits of RNA polymerase III and the three subunits of TFIIIB are differentially phosphorylated under normal versus repressing conditions. For these experiments, strains were constructed in which each pol III component was chromosomally epitope-tagged at its C-terminus (except for TBP, which was tagged at its N-terminus, S1 Table). Gene aliases are used in referring to polymerase subunits (rather than the standard naming convention) as this nomenclature allows easy recognition of the shared subunits. Growth curves measured in real time using a Bioscreen instrument yielded doubling times for most of the tagged strains that were comparable to the untagged parental strain (S2 Table). However, some tagged strains (C31, AC19, C17, C11 and ABC10α) showed a modest increase in doubling time. In the most extreme case, ABC23, the doubling time increased from 92 to 181 minutes (S2 Table). Next, lysates from vehicle or rapamycin-treated log phase cells were prepared and separated on SDS-polyacrylamide gels in the presence or absence of Phos-tag acrylamide which retards the migration of phosphorylated proteins [36]. In the absence of the Phos-tag reagent, western blotting showed a single band of the appropriate size for all subunits indicating that there was no detectable degradation of the proteins during sample preparation.

**Fig 2:**
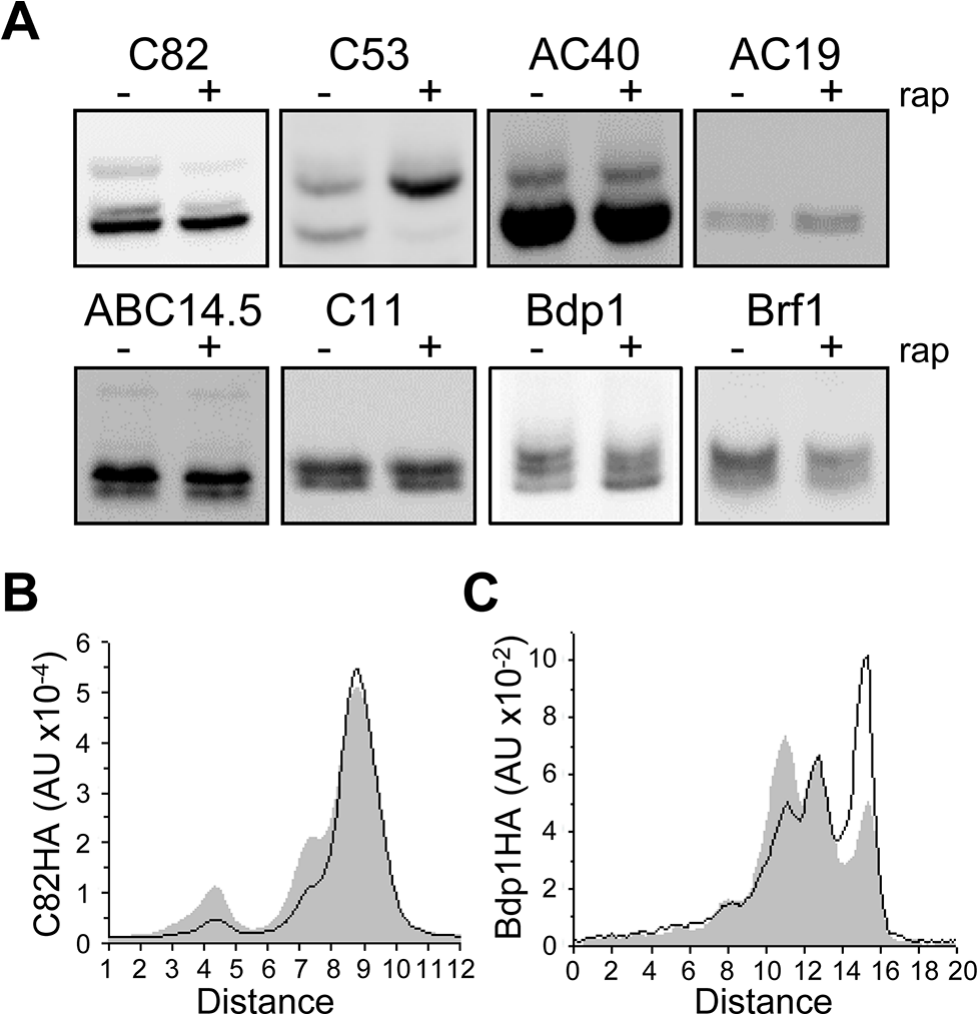
A subset of pol III and TFIIIB subunits are resolved into multiple bands by Phos-tag gel electrophoresis. **A.** Denatured extracts prepared from the indicated strains after treatment with vehicle or rapamycin were separated on Phos-tag gels for immunobloting using a HA monoclonal antibody. The acrylamide concentration required for optimal resolution of each protein into multiple bands was determined empirically and ranged from 6 to 15%. **B**. Differential migration of C82. A line peak profile of the C82HA signal from untreated extracts (solid gray) is superimposed on the line peak profile generated from rapamycin-treated extracts (solid black line). The amount of slow migrating species after rapamycin treatment is reduced. **C.** Differential migration of Bdp1. A line peak profile of the Bdp1HA signal from untreated extracts (solid gray) is superimposed on the line peak profile from rapamycin-treated extracts (solid black line). Note the shift from slow migrating species to fast migrating species with rapamycin treatment.

Phosphoproteomic studies have mapped at least one phosphosite in 15 of the 20 subunits that collectively comprise pol III and TFIIIB (S2 Table). In contrast, we identified only eight subunits that had two or more bands in Phos-tag gels. This difference is likely due to several factors (see Discussion) including the high sensitivity of contemporary mass spectrometry which can identify low stoichiometry phosphosites that are below the detection limit of Phos-tag gels and western blotting. Consistent with a low stoichiometry of phosphorylation, only six pol III subunits are reported phosphoproteins based on in vivo ^32^P-labeling (C82, C53, C31, AC40, AC19 and ABC23) [10, 45] although phosphoproteomic data are available for 12 of the 17 pol III subunits (S2 Table, Fig 2A and S1A Fig). Also, as growth in low phosphate media (required for in vivo labeling) causes low pol III transcription, some of the phosphorylations (e.g. C53)[10], see below) may have inhibitory function. Four pol III subunits (AC19, AC40, C53 and C82) were resolved into multiple bands by Phos-tag gel analysis.

In previous work, we identified three functionally important sites in Rpc53 that are phosphorylated to high stoichiometry under repressing conditions [10]. Consistent with the concerted phosphorylation of these sites by the kinases, Kns1 and Mck1, we observed two C53 bands in Phos-tag gels, one dephosphorylated species and one likely corresponding to the triply phosphorylated species that predominates after rapamycin treatment (Fig 2) [10]. We infer that the other 11 phosphosites identified in C53 by mass spectrometry represent minor species under normal or repressing conditions. Interestingly, we detected three forms of C82 under optimal growth conditions and found that the two slower migrating forms decreased in relative abundance after rapamycin treatment (Fig 2A and 2B). The relative loss of these bands in treated extracts was reproduced in extract titrations and replicated with independent samples. Thus, C82 appears to be differentially phosphorylated. Curve fitting of the peak intensities indicated that the phosphorylation state of ~20% of the total C82 signal was changed in response to rapamycin (S1B Fig). Three other subunits, AC40, AC19 and ABC14.5, which are known to be phosphorylated (S2 Table), showed two bands on Phos-tag gels although their relative abundance did not change after rapamycin treatment (Fig 2A). A similar result was obtained for C11 although no phosphosites have been reported in this protein.

Phosphoproteomic studies have mapped phosphosites in all three subunits of TFIIIB (S2 Table). However, our Phos-tag gel analysis showed only a single band for TBP before or after treatment with rapamycin (S1A Fig). For Brf1, two bands were resolved although there was no change in their relative abundance in response to rapamycin. Interestingly, Bdp1 showed four bands in log phase cells and a significant change towards lower levels of phosphorylation in the presence of the drug (Fig 2A and 2C). Curve fitting of band intensities indicated a ~20% increase in the amount of fastest migrating, dephosphorylated (confirmed below) Bdp1 band (S1C Fig). Our further experiments in this report focused on Bdp1.

### Identification of the major phosphosites in Bdp1

**Fig 3:**
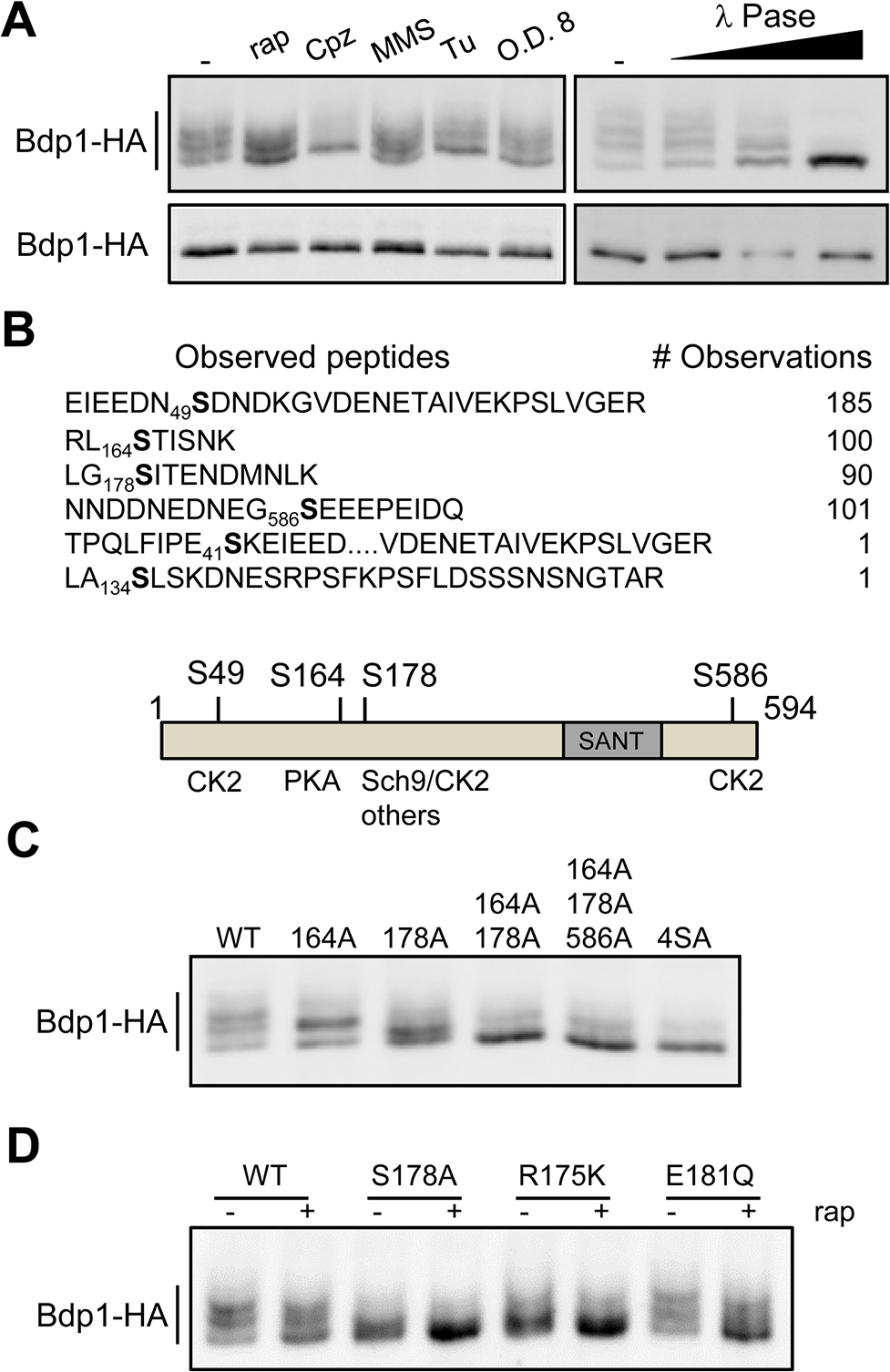
Bdp1 is a phosphoprotein that is dephosphorylated under stress conditions. **A.** Western analysis of Bdp1 phosphorylation. Denatured extracts prepared from a *BDP1-HA* strain treated with vehicle or various stressors were separated on Phos-tag gels (upper panels) and by standard SDS-PAGE (lower panels). Stressors include rapamycin (rap), chlorpromazine (cpz), methyl methanesulfonate (MMS), tunicamycin (Tu) and growth to stationary phase (O.D. 8) (left panel). An extract from logarithmically growing cells containing Bdp1HA was treated with 0, 1, 10 or 100 Units of λ phosphatase for 1h at 30°C (right panel). **B.** Major Bdp1 phosphopeptides reported by mass spectrometry studies [48]. Each phosphorylation site is annotated by amino acid position and presented in the context of the observed peptide and its total number of observations. A schematic of the major Bdp1 phosphosites is depicted underneath along with the kinase predicted by the consensus site at each location. **C.** Mutagenesis of Bdp1 phosphosites. Single and multiple alanine substitutions at the major phosphosites in Bdp1 (panel B) were analyzed for an effect on Bdp1 migration by Phos-tag gel electrophoresis. **D**. Phos-tag gel analysis of mutants that disrupt the Sch9 motif (R175K) and CK2 motif (E181Q) in Bdp1.

A hallmark of the known phosphoregulated components in the yeast pol III system (Maf1 and C53) is that their differential modification is not limited to a single repressing condition but is observed under multiple (all tested) conditions. Ubiquitous responses such as these are not, *a priori*, required to implicate a protein in a regulatory process. However, when the same effect is seen under diverse conditions, it is clearly more likely that the modification is of fundamental importance. Accordingly, the conversion of slower migrating forms of Bdp1 into faster migrating forms on Phos-tag gels was seen not only after rapamycin inhibition of TORC1, (which signals nitrogen and amino acid starvation [46]) but under other known pol III repressing conditions, such as plasma membrane stretching (CPZ), alkylating DNA damage (MMS), ER stress (Tu), and growth to high OD (low glucose) (Fig 3A, upper left panel). These changes are not due to the degradation of Bdp1 since the protein migrates as a single band on regular SDS-PAGE (Fig 3A, lower left panel). Moreover, in response to treatment with lambda phosphatase, the slow migrating forms of Bdp1 in a log phase cell extract were quantitatively converted into a single fast migrating form (Fig 3A, right panels). We conclude that Phos-tag gel analysis resolves multiple phosphorylated Bdp1 proteins in log phase cell extracts and that the protein is dephosphorylated to a variable degree by different repressing conditions (see S2A Fig for line peak profile comparisons).

Previous mass spectrometry studies have identified six phosphosites in Bdp1 (Fig 3B, S2 Table). We postulated that sites in frequently observed phosphopeptides are most likely targeted under optimal growth conditions and selected S49, S164, S178, and S586 for single and multiple alanine-substitution mutagenesis. Interestingly, each of these sites occurs at a consensus motif for a pro-growth kinase that is known to regulate pol III transcription; positions S49 and S586 conform to CK2 recognition motifs, position S164 conforms to a PKA consensus motif and the site at S178 has the potential for redundant phosphorylation by several kinases (Fig 3B). Phos-tag gel analysis of cell extracts from the mutant strains showed that single S164A and S178A mutations each produced a qualitative change in the banding pattern that reflects reduced phosphorylation (Fig 3C and S2B Fig). The double alanine mutant, S164/S178, was even more dramatically affected with the majority of the protein being dephosphorylated. Further mutagenesis to generate a quadruple 4SA mutant had little additional effect on Bdp1 mobility suggesting that the phosphorylation of S49 and S586 involves only a small fraction of the total Bdp1 protein. We also explored the contribution of CK2 to phosphorylation of S178. This residue is not only located in a consensus motif for CK2 but also contains Arg at position -3, a specificity residue for many yeast kinases including Sch9 [47]. Mutagenesis of the Arg residue (R175K) and the CK2 motif (E181Q) generated alleles that limit the phosphorylation of S178 to CK2 and kinase(s) with selectivity for Arg at position 175, respectively. Phos-tag gel analysis of untreated cell extracts containing these Bdp1 substitutions showed that the R175K mutation collapsed Bdp1 mobility almost as effectively as the S178A mutation whereas the E181Q mutation did not have a significant effect (Fig 3D). This suggests that in logarithmically growing cells S178 in bulk Bdp1 is predominantly targeted by a kinase(s) that recognizes the RxxS motif.

### PKA, Sch9 and CK2 Phosphorylate Bdp1 *in vitro*

**Fig 4:**
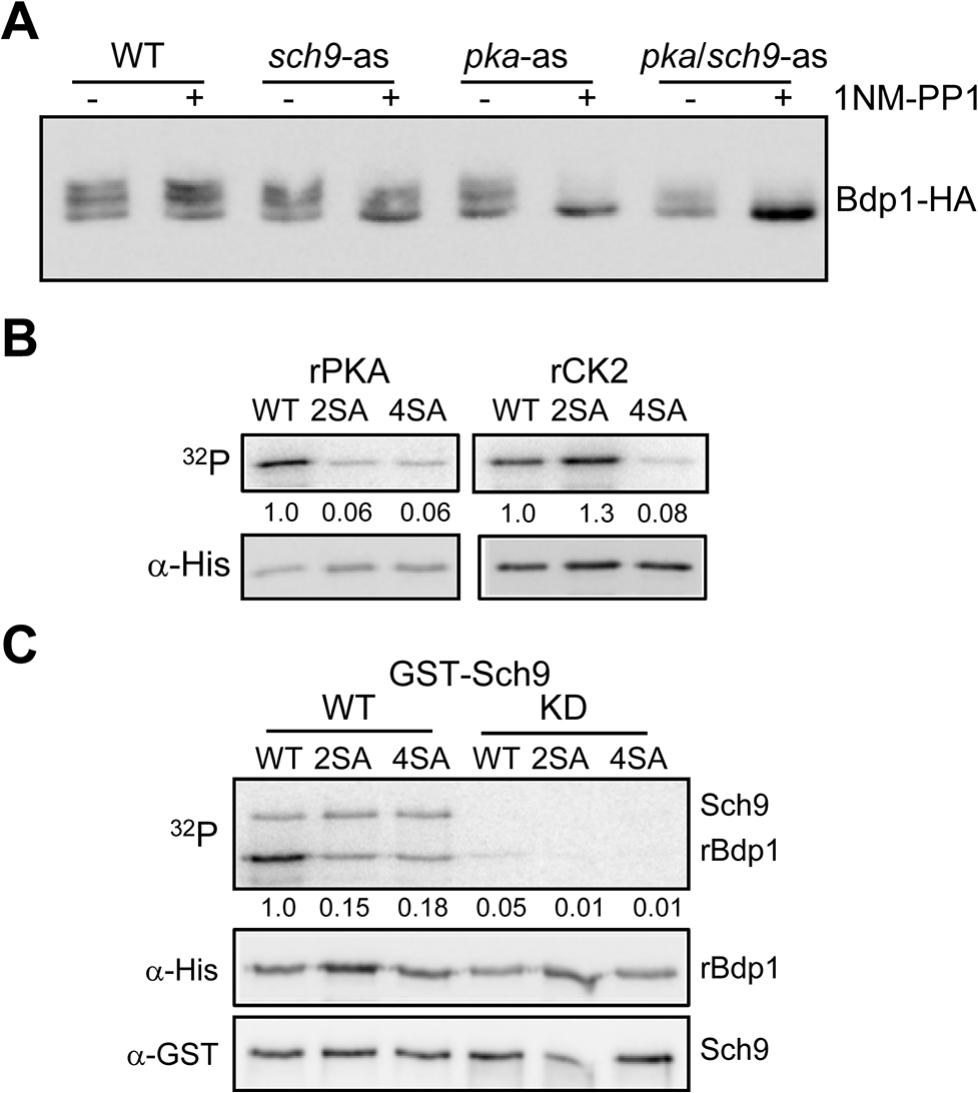
Bdp1 is phosphorylated by PKA, Sch9 and CK2 kinases. **A.** Wild-type and analog-sensitive *pka-as*, *sch9-as* and *pka-as sch9-as* strains that contained Bdp1HA were grown to mid-log phase and treated with 0.1 μM 1 NM-PP1 or drug vehicle (DMSO) for 1 hour before extract preparation. Bdp1HA was resolved in Phos-tag gels and detected by immunoblotting. **B.** Bdp1 is phosphorylated in vitro by PKA and CK2. Recombinant wild type (WT) and alanine-substituted Bdp1-6His 2SA (S164A and S178A) and 4SA (S49A, S164A, S178A and S586A) proteins were labeled in vitro by murine PKA (left panels) and human CK2 (right panels). The extent of phosphorylation (top panels) normalized to input protein detected by immunoblotting (lower panels) is reported under each lane. **C**. Bdp1 is phosphorylated in vitro by yeast Sch9. Recombinant wild-type (WT) and alanine-substituted Bdp1-6His 2SA and 4SA proteins were labeled in vitro with yeast-purified wild-type (left panels) and kinase-dead (KD, right panels) GST-Sch9. Phosphorimage (top panel) shows autophosphorylation of the catalytically active Sch9 and labeling of rBdp1. The extent of phosphorylation (normalized to input rBdp1, middle panel) is reported under each lane. GST-Sch9 proteins, detected by western, are shown in the lower panel.

The location of Bdp1 phosphosites within consensus motifs for well-known pro-growth kinases, PKA, Sch9 and CK2, coupled with the dephosphorylation of Bdp1 under repressing conditions, suggested that Bdp1 function might be regulated by these enzymes. Accordingly, reducing the activities of the kinases in vivo should lead to lower levels of Bdp1 phosphorylation. To examine this possibility for PKA and Sch9, Bdp1 was HA-tagged in the analog-sensitive strains (see Fig 1) and the effect of 1NM-PP1 on Bdp1 mobility was assessed using Phos-tag gels. Loss of Sch9 activity by analog treatment of the *sch9-as* strain increased the relative amount of dephosphorylated Bdp1. Analog treatment of the *pka-as* and *pka/sch9-as* strains caused almost complete dephosphorylation of Bdp1 (Fig 4A). Since only one of the identified Bdp1 phosphosites conforms to the PKA consensus, the results suggest that inhibiting PKA activity may have both direct and indirect effects on Bdp1 phosphorylation.

In vitro kinase assays were used to show that all three kinases are capable of phosphorylating Bdp1. Recombinant PKA readily phosphorylated wild-type recombinant Bdp1 in vitro (Fig 4B). This phosphorylation was reduced by more than 90% in a Bdp1 4SA mutant that lacked the four major phosphorylation sites. Mutation of the PKA and Sch9 sites (residues S164 and S178) in the 2SA mutant also reduced phosphorylation by PKA by more than 90% (Fig 4B, left panel). Since S164 conforms to a PKA consensus sequence, these results suggest that this residue is phosphorylated by PKA. Phosphorylation of wild-type recombinant Bdp1 by recombinant CK2 was dependent on a separate subset of the major phosphosites (Fig 4B, right panel). The Bdp1 4SA protein was a poor substrate for CK2. However, CK2 phosphorylated the Bdp1 2SA mutant protein as efficiently as wild-type indicating that S49 and/or S586 are targeted by CK2. Yeast-purified Sch9, but not the kinase-dead protein, phosphorylated Bdp1 in vitro (Fig 4C). Neither the 2SA nor the 4SA phosphosite mutants of Bdp1 were efficient substrates for Sch9 phosphorylation indicating the significance of S164 and/or S178 and the Sch9 recognition motif at these sites. Together, these in vitro findings support the identification of Bdp1 as a substrate for PKA, Sch9 and CK2 kinases in vivo.

### The phosphosite mutant *BDP1-4SA* is hyper-repressible in a sensitized *MAF1myc* strain

**Fig 5:**
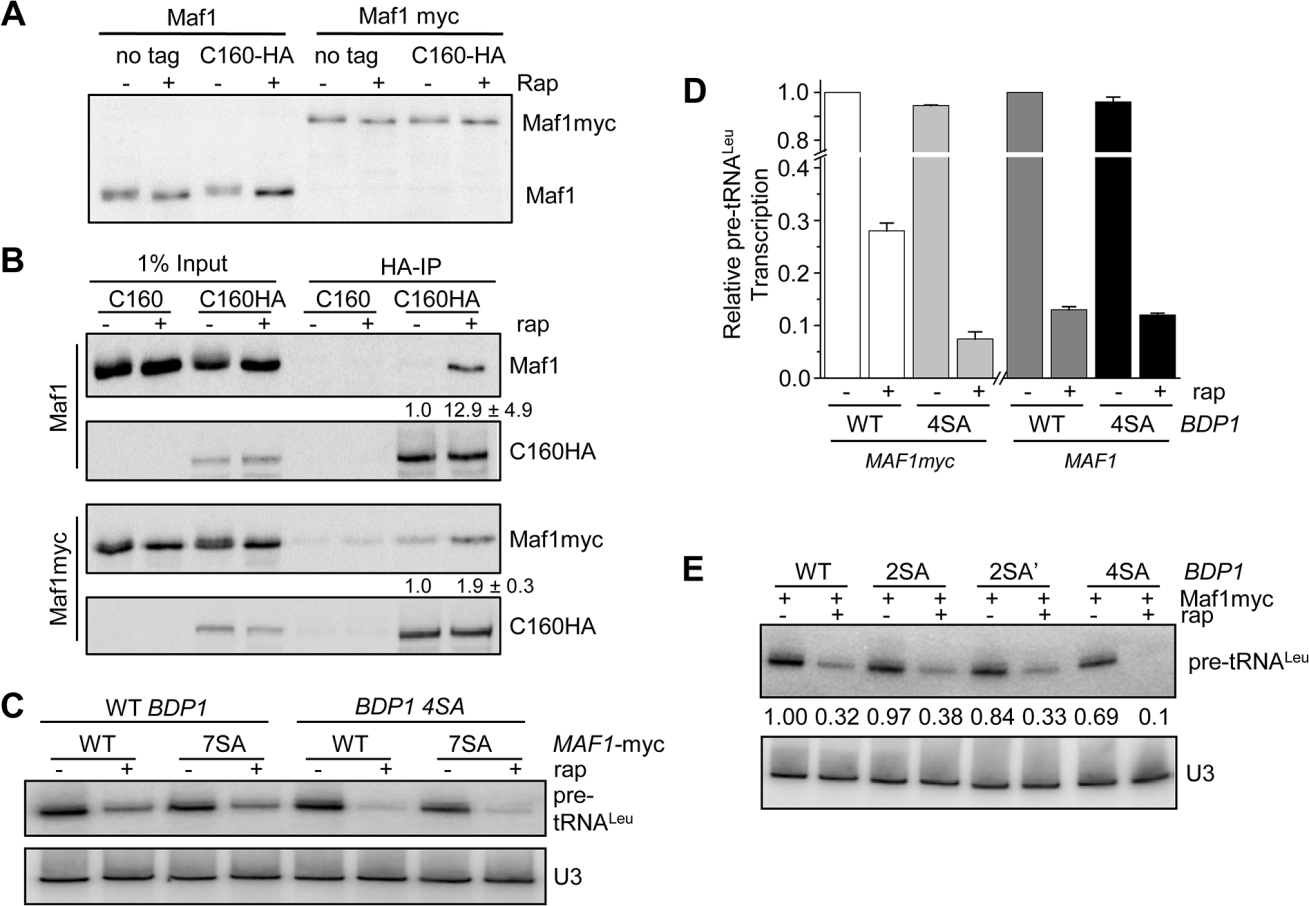
Bdp1 phosphorylation state affects pol III transcription. **A.** Maf1 and Maf1myc are expressed at similar levels in cell extracts. Western blotting of Maf1 and Maf1myc was performed with a polyclonal Maf1 antibody in extracts of untagged or Rpc160-HA tagged strains that had been treated or not with rapamycin. Band quantitation was by line peak integration as described in Materials and Methods. **B.** Maf1 and Maf1myc proteins differ in their apparent binding affinity for Rpc160. Rpc160-HA immunoprecipitations were performed on the extracts shown in panel A. Maf1 and Maf1myc proteins that co-immunoprecipitate with Rpc160-HA were detected by immunoblotting with Maf1 polyclonal and α-myc antibodies, respectively. Rpc160-HA was detected using an α-HA antibody. The blots are representative of independent replicate experiments. The fold increase in co-immunoprecipitated Maf1 or Maf1myc in the rapamycin-treated samples represents the mean ± standard deviation and is shown below the respective panels. **C.** Pol III transcription in *BDP1-4SA* strains is hyper-repressed after rapamycin treatment. Northern analysis of pol III transcription in strains containing wild-type *BDP1* or *BDP1-4SA* and either wild-type *MAF1myc* or *MAF1myc-7SA*. Strains were grown to mid-log phase and treated with rapamycin or drug vehicle (DMSO) for 1 hour before RNA preparation. **D.** Quantitation of experiments comparing transcriptional repression in wild-type untagged *MAF1* (dark grey and black bars) and *MAF1myc* (white and light grey bars) strains in the context of wild-type *BDP1* or *BDP1-4SA*. Each bar in the histogram represents the mean ± SEM of biologically independent triplicate experiments. **E.** *BDP1-4SA* is required for enhanced repression by rapamycin. Pol III transcription before and after rapamycin treatment was assessed in *MAF1myc* strains containing wild-type *BDP1*, *BDP1*-*2SA* (S164/S178A), *BDP1-2SA’* (S49/S586A) or *BDP1-4SA*. The amount of pre-tRNA^Leu^ transcript normalized to U3 snRNA is expressed relative to the untreated wild-type strain and is indicated below each lane.

The preceding observations together with published data show that (i) loss of phosphorylation of Bdp1 correlates with loss of pol III transcription under cellular stress (Figs 2A, 2C and 3A); (ii) Bdp1 phosphorylation in vitro by three pro-growth kinases occurs at consensus sites that are highly represented in yeast phosphoproteomic data (Figs 3B, 4B and 4C) [48] and (iii) inhibition of these kinases in vivo correlates with loss of Bdp1 phosphorylation and repression of pol III transcription (Figs 1A and 4A). These findings suggest that phosphosite mutants of Bdp1 are likely to differentially affect pol III transcription and/or its regulation. Accordingly, *BDP1* mutants were prepared with all four phosphosites mutated to alanine (*BDP1-4SA*) or glutamic acid (*BDP1-4SE*) and pre-tRNA^Leu^ synthesis was compared to wild-type *BDP1* in control and rapamycin-treated cells by northern analysis. In these experiments, neither *BDP1* mutant affected the amount of pre-tRNA^Leu^ in untreated cells nor the level of transcriptional repression in response to rapamycin (S3 Fig). The results of triplicate experiments for wild-type *BDP1* and *BDP1-4SA* are presented graphically in Fig 5D (dark grey and black bars).

During the course of this study, it became apparent that the extent of repression of pol III transcription was different in strains containing untagged *MAF1* versus a C-terminal myc-tagged *MAF1* allele. From a total of 23 independent experiments in an otherwise wild-type strain background, we found that a one hour treatment with rapamycin reduced transcription in the untagged *MAF1* strain to 18 ± 2% (SEM, n = 17) of the untreated control versus only 32 ± 5% (SEM, n = 6) in the *MAF1myc* strain (*p* = 0.0025, Student’s t-test). This suggested that the C-terminal myc epitope compromised the effectiveness of Maf1 as a repressor. Similar results were obtained in a *BDP1-HA* strain. In this case, rapamycin reduced the amount of pre-tRNA^Leu^ in the untagged *MAF1* strain to 11.7 ± 0.3% (SEM, n = 3, e.g. Fig 5D and S3A Fig) of the untreated control versus 29.3 ± 1.3% (SEM, n = 3, e.g. Fig 5D and S3B Fig left-most two lanes) in the *MAF1myc* strain.

We next asked if the association of Maf1 with pol III, which is known to increase under repressing conditions [5, 49, 50], was also negatively affected when Maf1 was myc-tagged at its C-terminus. We compared the efficiency of Maf1 and Maf1myc co-immunoprecipitation with Rpc160 in control and rapamycin-treated cell extracts [37]. Maf1 and Maf1myc were expressed at similar levels in the respective lysates and Rpc160 was equally well recovered from treated and untreated Maf1 and Maf1myc strains, as measured by immunodetection of the HA epitope (Fig 5A and 5B). Rapamycin treatment increased the amount of untagged Maf1 associated with Rpc160 approximately 13-fold whereas the association of Maf1myc increased less than 2-fold (Fig 5B). This weaker binding of Maf1myc to Rpc160 was not affected by the Bdp 4SA mutation (S4 Fig). Together these data suggest that the myc epitope limits the association of Maf1 with pol III upon cellular stress consistent with the reduced ability of Maf1myc to repress transcription.

We used the lower apparent affinity of Maf1myc for pol III to reevaluate the link between Bdp1 phosphorylation and transcription. Wild-type *BDP1*, *BDP1*-*4SA* and *BDP1-4SE* strains deleted for *MAF1* were transformed with plasmids containing wild-type *MAF1myc* or mutant *MAF1myc* alleles (i.e. the non-phosphorylatable 7SA and the phosphomimetics 6SE and 7SE) in an effort to promote or antagonize repression, respectively. Weakening of the interaction between Maf1myc and pol III using phosphomimetic *MAF1* mutants (6SE and 7SE)[4, 5] did not uncover any capacity of the *BDP1-4SE* mutant to increase transcription in log phase cells or attenuate repression by rapamycin (S3B Fig). In contrast, the combination of wild-type *MAF1myc* and *BDP1-4SA* resulted in significantly greater repression of transcription compared to its wild-type *BDP1* control (Fig 5C and 5D, 7.9 ± 1.2% versus 29.3 ± 1.3% residual transcription, ± SEM). This hyper-repression phenotype was not further enhanced in the presence of *MAF1myc-7SA* (Fig 5C). Thus, the weakened capacity of Maf1myc in repression exposed the effect of a constitutively non-phosphorylatable Bdp1 protein in regulating transcription. In a subsequent experiment, we examined the contribution of the PKA/Sch9 sites at S164 and S178 and the CK2 sites at S49 and S586 towards the hyper-repressible phenotype by separately mutating each pair of sites (*BDP1-2SA* and *BDP1-2SA’*, respectively). These double mutants showed levels of repression comparable to wild-type *BDP1* in the *MAF1-myc* background; no hyper-repression of transcription was achieved, unlike the quadruple mutant (Fig 5E). Thus, the results suggest that loss of phosphorylation at all four sites in Bdp1 is necessary to cause hyper-repression of transcription in the sensitized strain background and support the in vivo and in vitro data that PKA, Sch9 and CK2 regulate Bdp1 phosphorylation to promote pol III transcription.

## Discussion

We screened all 17 pol III subunits and the three TFIIIB subunits for evidence of differential phosphorylation using Phos-tag SDS-PAGE. A relatively high stoichiometry of phosphorylation was observed for the subunit ABC14.5, common to all three polymerases, the AC40 and AC19 subunits shared between pols I and III, the pol III-specific subunits, C82, C53 and C11 and the TFIIIB factors, Brf1 and Bpd1 (Fig 2). Three proteins were found to be differentially modified: C82, C53 and Bdp1.

The Phos-tag SDS-PAGE screening technique resolved multiple bands for some, but not all, proteins identified as phosphoproteins by high throughput mass spectrometry (summarized in S2 Table). This difference may be due to both technical and biological factors. We found that the resolution of multiple bands for any individual protein was dependent on the method of sample preparation, the acrylamide concentration and the amount of extract loaded. For example, the ability to resolve multiple phosphorylated species of Bdp1 in Phos-tag acrylamide gels appeared to be limited by in-gel competition with co-migrating phosphoproteins since the phosphorylated Bdp1 forms were lost at higher protein loads. In addition, as noted previously, some proteins may be phosphorylated at very low levels that are detectable by mass spectrometry but fall below the sensitivity of Western detection used in our screen. Of the eight proteins exhibiting multiple species in Phos-tag gels, only three were found to be differentially modified in response to inhibition of TORC1, a treatment that represses transcription by pols I and III and affects a significant fraction of the pol II transcriptome. Notably, studies on yeast pol I have shown that not all high stoichiometry phosphosites are regulated, surface accessible in the final complex or essential for polymerase function in vivo [51]. A similar situation may apply for pol III. In addition, some regulatory modifications in this system may target the small fraction of pol III and TFIIIB molecules bound to DNA or actively engaged in transcription (fewer than 500 molecules of each per cell). Lastly, it should be noted that the screening technique used here would not report differential phosphorylation if the phosphosites changed but total protein phosphorylation was preserved.

The screen confirmed the phosphoregulation of C53, known to be targeted by Kns1 and Mck1 kinases upon cell stress [10] and identified a second differentially phosphorylated pol III subunit, C82. In contrast to C53, the relative abundance of slow migrating C82 bands decreased on rapamycin treatment indicating that loss of C82 phosphorylation occurs on inhibition of TORC1 signaling (Fig 2A and 2B). C82 is a component of the C82/C34/C31 TFIIE-like subcomplex specific to pol III and contributes to open complex formation and to the recruitment of pol III by Brf1 [52–54]. Cryo-EM studies show that the position of the C82/C34/C31 subcomplex is rearranged when the repressor, Maf1, is bound to pol III [8]. Photocrosslinking studies, coupled with structural modeling, position the C82/34/31 subcomplex around the pol III active center cleft to potentially influence open promoter complex formation and stabilization [55]. Thus, loss of C82 phosphorylation could promote repositioning of the C82/C34/C31 subcomplex and/or Maf1 binding to favor the subsequent inhibition of transcription.

Phosphoproteomic data indicate that all three subunits of TFIIIB are phosphoproteins. TBP is a known substrate of CK2 in vitro [56] and loss of CK2-mediated TBP phosphorylation has been linked to the regulation of pol III transcription in response to DNA damage [25]. The present experiments did not provide any indication that TBP is differentially phosphorylated in response to rapamycin as only a single band was observed. In contrast, multiple Brf1 bands were identified but their distribution did not change with rapamycin treatment. Thus, the possibility of opposing phosphorylations at different sites remains open. Our studies found that Bdp1 is differentially modified and determined that the important sites correspond to four residues that are frequently observed in phosphoproteomic data (Fig 3, S2 Table). These sites map to consensus motifs for three pro-growth kinases, CK2, PKA and Sch9, all of which are known to regulate pol III transcription [3]. Each of these kinases phosphorylated Bdp1 at the appropriate sites in vitro (Figs 3B, 4B and 4C). Although the PKA and Sch9 sites at S164 and S178 contribute to the bulk of the phospho-differential seen on rapamycin treatment, the CK2 sites at S49 and S586 contribute to Bdp1 function. The quadruple alanine-substituted *BDP1-4SA* mutant was the only *BDP1* mutant that had a transcription phenotype in our study; enhanced repression in a sensitized *MAF1myc* strain background. Thus, the data indicate that phosphorylation of Bdp1 at these sites opposes Maf1-mediated repression.

The potential for phosphorylation of Bdp1 at S178 by Sch9 (or other Arg-3 specificity kinases) and CK2 suggests a level of redundancy for modification at this position. Sch9 could phosphorylate Bdp1 in the cytoplasm before its nuclear import and/or it may act on Bdp1 in the nucleus although Sch9 is primarily a cytoplasmic protein [35]. In the nuclear compartment, phosphorylation of S178 in Bdp1 could be accomplished by different kinases depending on whether the factor is bound or not to DNA. The amount of Bdp1 bound in TFIIIB-DNA complexes is only a small fraction of the total (~5%)[57]. Consequently, mutations that alter its phosphorylation only in the context of chromatin may not be detected in total cellular Bdp1. Thus, a role for CK2 in S178 phosphorylation cannot be excluded simply by mutation of the CK2 motif (E181Q). A different approach will be needed to address this question.

In yeast, CK2 activity is required for high levels of pol III transcription in vitro [24] and in vivo [25], the kinase is associated with tRNA gene loci and alkylating DNA damage decreases the amount of CK2 activity in TBP immunoprecipitates [25–27]. However, the mechanism controlling CK2 activity or function in response nutrients and stress has been unclear as the enzyme is not part of known signaling pathways and is thought to be constitutively active [58, 59]. Recent work has found that the Ckb1 regulatory subunit of CK2 is phosphorylated by the TORC1-regulated kinase Kns1 under various stress conditions including treatments with rapamycin and methyl methanesulfonate. Moreover, the phosphorylation of Ckb1 correlates with the reduced occupancy of CK2 at tRNA gene loci and repressed transcription [60]. These findings are consistent with the idea that phosphoregulation of Bdp1 by CK2 is controlled in the context of the TFIIIB-DNA complex which is stable during logarithmic growth and under acute repressing conditions in yeast [61, 62].

**Fig 6:**
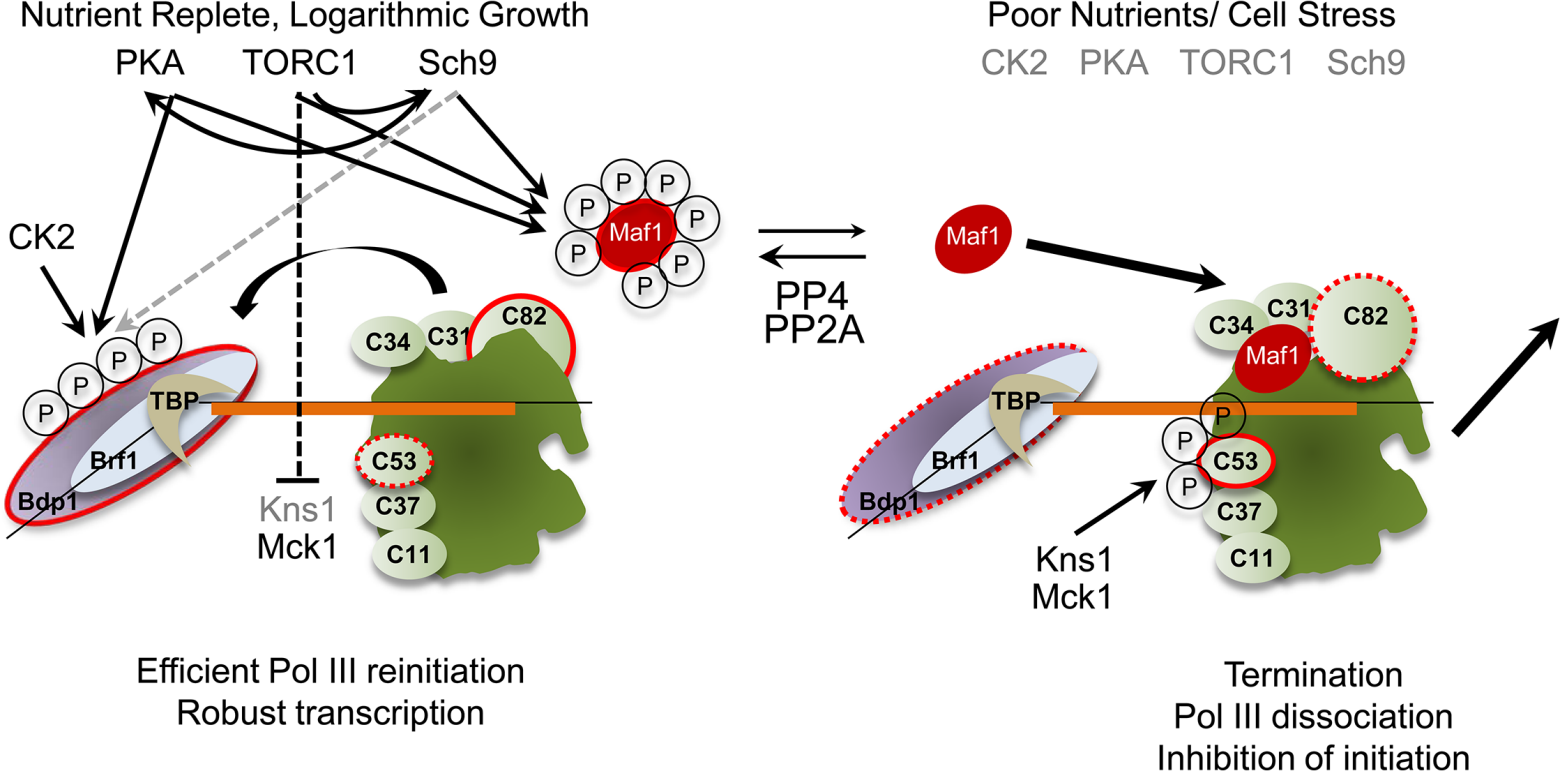
A model of the regulation of pol III transcription in response to nutrients and stress. Logarithmic growth in nutrient replete conditions and signaling by pro-growth kinases leads to phosphorylation of the TFIIIB subunit, Bdp1, the pol III subunit C82 and the repressor Maf1. The dephosphorylation of these proteins in poor nutrients and under stress is accompanied by C53 phosphorylation by the stress-regulated kinase Kns1 and Mck1. Additional details are provided in the text. TFIIIB and pol III subunits that are differentially phosphoregulated are outlined in red. Solid red outlines indicate phosphorylation while dashed red outlines indicate loss of phosphorylation. Known regulatory phosphorylation sites are indicated by the letter P enclosed in a circle. The dashed line connecting TORC1 to Kns1 indicates that kinase expression is low under optimal growth conditions [10]. The gray dashed line indicates that the action of Sch9 on Bdp1 may occur in the cytoplasm before nuclear import. Note that kinases other than Sch9 may target S178. While the phosphatases PP2A and PP4 have been documented to affect pol III transcription (reviewed in [3]), the phosphatase(s) that dephosphorylate C82 and Bdp1 have yet to be identified.

The differential modification of C82 and Bdp1 reported here provides additional links between the pol III transcription machinery and nutrient and stress signaling pathways in yeast. These modifications and the functional changes that ensue appear to work in an integrated manner in order to achieve the desired transcriptional output. This is suggested by the fact that phosphosite mutants of Bdp1, C53 and Maf1 have little to no effect on transcription by themselves and must be assayed in appropriately sensitized strain backgrounds to expose their functional consequences. The new findings have been incorporated into an evolving model [3, 10] for the regulation of pol III transcription in response to cellular signaling (Fig 6). Previous work has demonstrated that Maf1-dependent repression of pol III transcription occurs after the polymerase dissociates from the template; Maf1 can interact with the elongating polymerase but nucleotide polymerization is not inhibited whereas the polymerase-Maf1 complex in solution cannot initiate transcription [3, 5, 7, 8, 37]. Moreover, pol III molecules undergoing facilitated recycling, i.e. the polymerase reinitiates on the same template that it has just transcribed without dissociation [63, 64], are resistant to repression by Maf1 [7]. As discussed previously, this suggests that facilitated recycling needs to be interrupted to enable efficient Maf1-dependent repression [3, 7, 10]. We propose that the differential phosphorylation of pol III components is intended to achieve this outcome. Thus, phosphorylation of Bdp1 in TFIIIB and of C82 in pol III is predicted to contribute to efficient rebinding of pol III to TFIIIB-DNA complexes during facilitated recycling to allow robust transcription. Conversely, loss of Bdp1 and C82 phosphorylation caused by poor nutrients and cell stress, coupled with phosphorylation of C53 by stress-activated kinases, is expected to limit pol III rebinding (e.g. due to reduced affinity for TFIIIB) and to promote pol III dissociation at the terminator [10], respectively. Maf1 in its dephosphorylated state efficiently binds to the elongating polymerase prior to its dissociation from DNA or captures the polymerase after dissociation to inhibit further initiation. We note that the model described above is not specific about how Bdp1 phosphoregulation may affect pol III recruitment and does not exclude effects on its interactions with Tfc4, Brf1, Sub1, Hmt1 or Isw2 [65–69]. Since the pol III machinery and the signaling molecules and pathways that regulate its function are conserved in higher eukaryotes, we anticipate that regulatory mechanisms comparable to those reported here will also operate in higher eukaryotes.

## Supporting Information

S1 Fig A subset of Pol III and IIIB subunits show no apparent phosphorylation. **A.** Western analysis of selected tagged pol III and TFIIIB subunits. Denatured extracts were prepared from strains treated with vehicle or rapamycin and separated by Phos-tag gel electrophoresis. Each protein was analyzed at several acrylamide concentrations (from 6 to 15%) but showed only a single band in extracts from these strains. **B.** Curve fits for C82. Western signal shown in Fig 2A was digitally detected by enhanced chemiluminescence (LAS400, GE) and analyzed in OriginPro 8.1. Left and right panels show representative curve fits for untreated and rapamycin-treated C82-HA extracts, respectively. Peaks are numbered from left to right. **C.** Curve fits for Bdp1. Extracts from *BDP1-HA* strains were separated and processed as described above. Left and right panels show representative curve fits for untreated and rapamycin-treated Bdp1 extracts, respectively.
(PDF)

S2 Fig Bdp1 mutations and transcription. **A.** Pol III transcription and the extent of repression by rapamycin is compared in untagged *MAF1* strains containing wild-type *BDP1* or *BDP1-4SA* expressed form their normal chromosomal locus. Strains were grown to mid-log phase and treated with rapamycin or drug vehicle (DMSO) for 1 hour before RNA preparation and northern analysis. Pre-tRNALeu levels are expressed relative to the untreated wild-type strain. Quantitation represents the average of two independent experiments. **B.** Pol III transcription and the extent of repression by rapamycin in untagged *MAF1* strains that are chromosomally deleted for *BDP1* and contain wild-type *BDP1* or *BDP1-4SA* expressed from a pRS315-based plasmid. **C.** Pol III transcription and repression in wild type *BDP1* and *BDP1-4SE* strains that are chromosomally deleted for *MAF1* and contain *MAF1myc* WT, 6SE or 7SE alleles on a pRS314-based plasmid. Transcription and repression were detected and reported as in panel A.
(PDF)

S3 Fig Bdp1 peak profile patterns. **A.** Bdp1 phosphorylation patterns after nutrient and cellular stresses. The line peak profiles of Bdp1 phosphorylation in untreated and treated extracts (Fig 3A) were generated as in S1B Fig. The Bdp1HA signal from Bdp1 wild-type extracts (solid gray) is superimposed on the cumulative line peak profile derived for each treatment (solid black lines). **B.** The Bdp1 phosphorylation pattern is altered in alanine-substitution mutants. A line peak profile of the Bdp1HA signal from Bdp1 wild-type extracts (solid gray) is superimposed on the cumulative line peak profile from each Bdp1 mutant in Fig 3C (solid black lines). Note that the pattern of individual peaks is more complex than is described by the cumulative curve fit.
(PDF)

S4 Fig Bdp1 phosphorylation state does not affect the association of Rpc160 and Maf1. Bdp1Wt and 4SA proteins do not alter Maf1 co-immunoprecipitation with Rpc160 in control and rapamycin-treated cells. HA immunoprecipitations were performed on extracts from W303 *Bdp1-3HA*::*KanR maf1*::*natR C160-3HA*::*hphR* pRS313*Maf1-9myc* and W303 *Bdp-3HA 4SA*::*KanR maf1*::*natR C160-3HA*::*hphR* pRS313*Maf1-9myc* strains. Maf1myc proteins that co-immunoprecipitate with Rpc160-HA were detected by immunoblotting with α-myc antibodies. Rpc160-HA was detected using an α-HA antibody. Input Maf1myc signals were overexposed to enable visualization of the co-immunoprecipitated protein. The blots are representative of independent replicate experiments.
(PDF)

S1 Table Yeast Strains. (PDF)

S2 Table Summary of Yeast RNA Polymerase III and TFIIIB Phosphorylation. (PDF)
