# Supplementary figures and images for: Differential Phosphorylation of RNA Polymerase III and the Initiation Factor TFIIIB in Saccharomyces cerevisiae
Source: PLoS One. 2015 May 13;10(5):e0127225. doi: 10.1371/journal.pone.0127225 (PMC4430316; doi:10.1371/journal.pone.0127225)

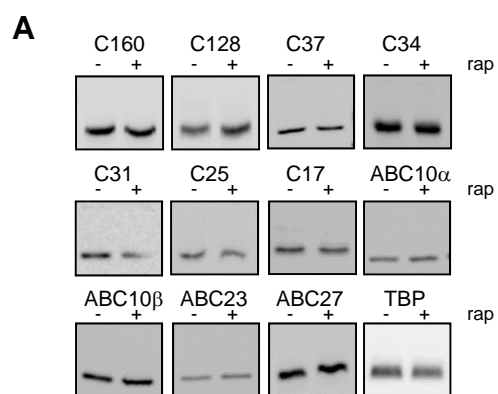

**B**

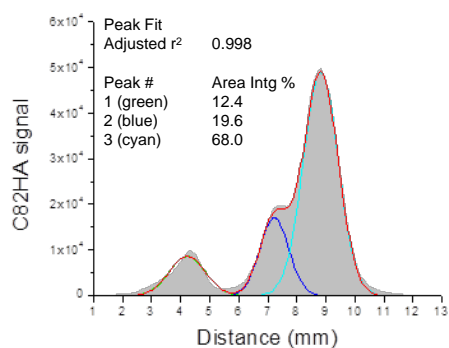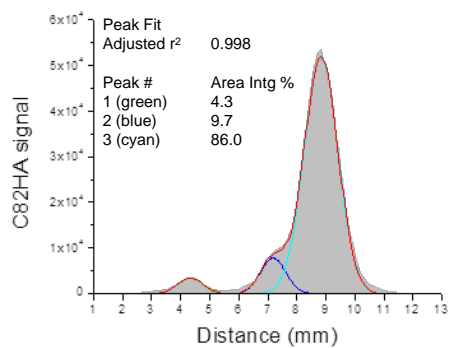

**C**

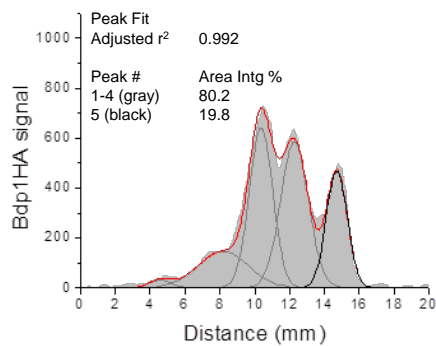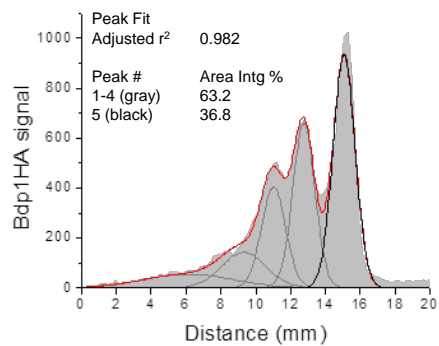

Supplement: S1 Fig — A. Western analysis of selected tagged pol III and TFIIIB subunits. Denatured extracts were prepared from strains treated with vehicle or rapamycin and separated by Phos-tag gel electrophoresis. Each protein was analyzed at several acrylamide concentrations (from 6 to 15%) but showed only a single band in extracts from these strains. B. Curve fits for C82. Western signal shown in Fig 2A was digitally detected by enhanced chemiluminescence (LAS400, GE) and analyzed in OriginPro 8.1. Left and right panels show representative curve fits for untreated and rapamycin-treated C82-HA extracts, respectively. Peaks are numbered from left to right. C. Curve fits for Bdp1. Extracts from BDP1-HA strains were separated and processed as described above. Left and right panels show representative curve fits for untreated and rapamycin-treated Bdp1 extracts, respectively. (PDF) [file pone.0127225.s001.pdf]

**A**

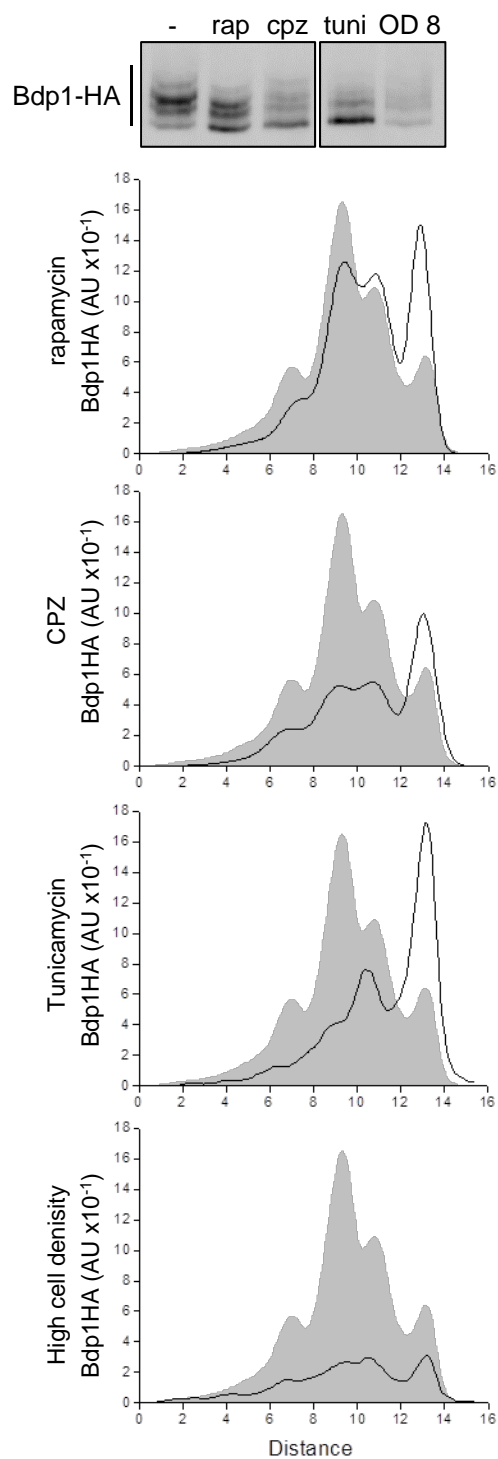

**B**

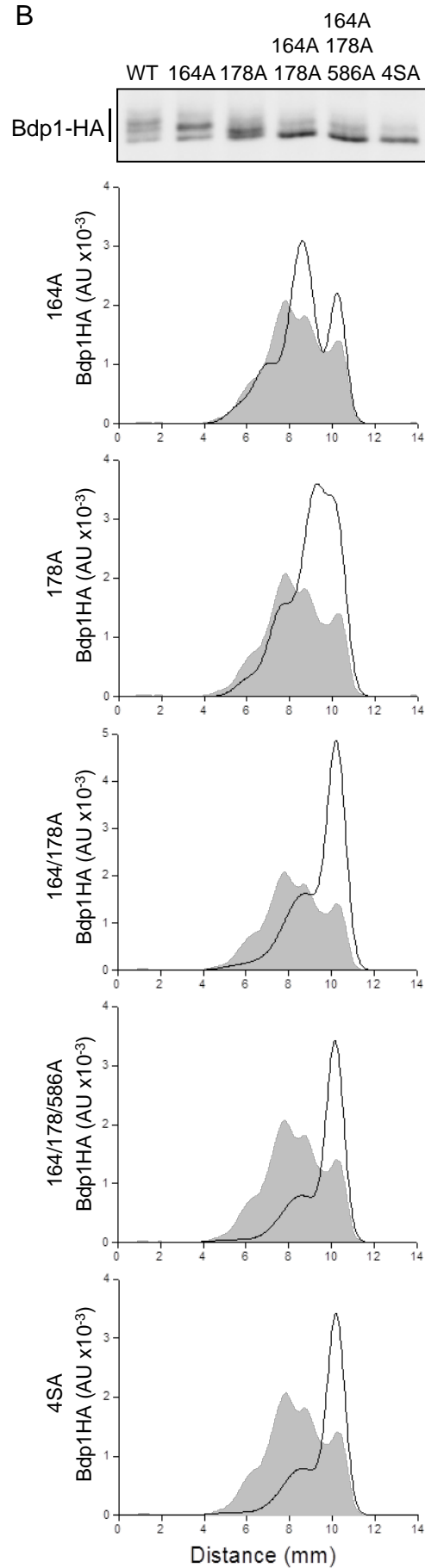

Supplement: S2 Fig — A. Pol III transcription and the extent of repression by rapamycin is compared in untagged MAF1 strains containing wild-type BDP1 or BDP1-4SA expressed form their normal chromosomal locus. Strains were grown to mid-log phase and treated with rapamycin or drug vehicle (DMSO) for 1 hour before RNA preparation and northern analysis. Pre-tRNALeu levels are expressed relative to the untreated wild-type strain. Quantitation represents the average of two independent experiments. B. Pol III transcription and the extent of repression by rapamycin in untagged MAF1 strains that are chromosomally deleted for BDP1 and contain wild-type BDP1 or BDP1-4SA expressed from a pRS315-based plasmid. C. Pol III transcription and repression in wild type BDP1 and BDP1-4SE strains that are chromosomally deleted for MAF1 and contain MAF1myc WT, 6SE or 7SE alleles on a pRS314-based plasmid. Transcription and repression were detected and reported as in panel A. (PDF) [file pone.0127225.s002.pdf]

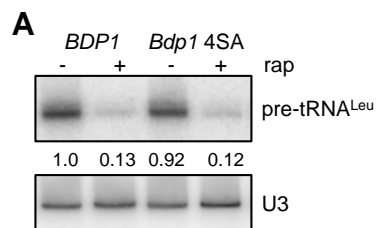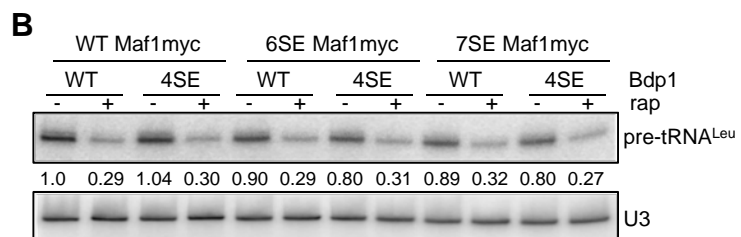

Supplement: S3 Fig — A. Bdp1 phosphorylation patterns after nutrient and cellular stresses. The line peak profiles of Bdp1 phosphorylation in untreated and treated extracts (Fig 3A) were generated as in S1B Fig. The Bdp1HA signal from Bdp1 wild-type extracts (solid gray) is superimposed on the cumulative line peak profile derived for each treatment (solid black lines). B. The Bdp1 phosphorylation pattern is altered in alanine-substitution mutants. A line peak profile of the Bdp1HA signal from Bdp1 wild-type extracts (solid gray) is superimposed on the cumulative line peak profile from each Bdp1 mutant in Fig 3C (solid black lines). Note that the pattern of individual peaks is more complex than is described by the cumulative curve fit. (PDF) [file pone.0127225.s003.pdf]

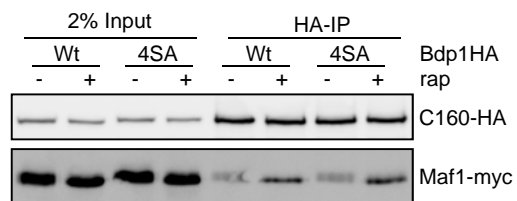

Supplement: S4 Fig — Bdp1Wt and 4SA proteins do not alter Maf1 co-immunoprecipitation with Rpc160 in control and rapamycin-treated cells. HA immunoprecipitations were performed on extracts from W303 Bdp1-3HA::KanR maf1::natR C160-3HA::hphR pRS313Maf1-9myc and W303 Bdp-3HA 4SA::KanR maf1::natR C160-3HA::hphR pRS313Maf1-9myc strains. Maf1myc proteins that co-immunoprecipitate with Rpc160-HA were detected by immunoblotting with α-myc antibodies. Rpc160-HA was detected using an α-HA antibody. Input Maf1myc signals were overexposed to enable visualization of the co-immunoprecipitated protein. The blots are representative of independent replicate experiments. (PDF) [file pone.0127225.s004.pdf]
