# Supplementary material for: Differential Phosphorylation of RNA Polymerase III and the Initiation Factor TFIIIB in Saccharomyces cerevisiae
Source: PLoS One. 2015 May 13;10(5):e0127225. doi: 10.1371/journal.pone.0127225 (PMC4430316; doi:10.1371/journal.pone.0127225)
Supplement: S1 Table — (PDF) [file pone.0127225.s005.pdf]

Table S1. Yeast strains

Parental strain	Additional mutations/Plasmids	Source[1]	Figure
BY4741	*RPC160-3HA::hphR*	this work	S1A
BY4741	*RPC128-3HA::hphR*	this work	S1A
BY4741	*RPC82-3HA::hphR*	this work	2A
BY4741	*RPC82-9myc::hphR RPC53-3HA::HIS3*	Lee et al., 2012	2A
BY4741	*RPC40-3HA:: hphR*	this work	2A
BY4741	*RPC37-3HA::hphR*	this work	S1A
BY4741	*RPC34-3HA::hphR*	this work	S1A
BY4741	*RPC31-3HA:: hphR*	this work	S1A
BY4741	*RPB5-3HA::hphR*	this work	S1A
BY4741	*RPC25-3HA::hphR*	this work	S1A
BY4741	*RPB6-3HA::hphR*	this work	S1A
BY4741	*RPC19-9myc::hphR*	this work	2A
BY4741	*RPC17-3HA::hphR*	this work	S1A
BY4741	*RPB8-3HA::hphR*	this work	2A
BY4741	*RPC11-9myc::hphR*	this work	2A
BY4741	*RPC10-9myc::hphR*	this work	S1A
BY4741	*RPB10-9myc::hphR*	this work	S1A
BY4741	*TDH3pr-3HA-TBP::natR*	this work	S1A
W303	*BRF1-3HA::HIS3*	Desai et al., 2005	2A
W303	*BDP1-3HA::kanR*	Desai et al., 2005	2A ,3A, 3C, 3D,4A
W303	*BDP1-3HA S164A::kanR*	this work	3C
W303	*BDP1-3HA S178A::kanR*	this work	3C,3D
W303	*BDP1-3HA S164A S178A::kanR*	this work	3C
W303	*BDP1-3HA S164A S178A S586A::kanR*	this work	3C
W303	*BDP1-3HA S49A S164A S178A S586A::kanR*	this work	3C
W303	*BDP1-3HA R175K::kanR*	this work	3D
W303	*BDP1-3HA E181Q::kanR*	this work	3D
W303	*pka-as BDP1-3HA::kanR*	this work	4A
W303	*sch9-as BDP1-3HA::kanR*	this work	4A
W303	*pka-as/shc9-as BDP1-3HA::kanR*	this work	4A
W303	*pka-as/sch-as maf1Δ::G418*	this work	1A
W303	*maf1Δ::G418* pRS314*Maf1-9myc*	Moir et al., 2006	1B
W303	*pka-as/sch9-as maf1Δ::G418* pRS314*MAF1-9myc*	Lee et al., 2009	1B
W303	*maf1Δ::G418* pRS314*Maf1-9myc 7SA*	Moir et al., 2012	1B
W303	*pka-as/sch-as maf1Δ::G418* pRS314*Maf1-9myc 7SA*	this work	1B
W303	*BDP1-3HA::kanR maf1Δ::natR* pRS313*MAF1-9myc*	this work	5C, 5D, 5E
W303	*BDP1-3HA::kanR maf1Δ::natR* pRS313*MAF1-9myc 7SA*	this work	5C
W303	*BDP1-3HA 4SA::kanR maf1Δ::natR* pRS313*MAF1-9myc*	this work	5C, 5D, 5E
W303	*BDP1-3HA 4SA::kanR maf1Δ::natR* pRS313*MAF1-9myc 7SA*	this work	5C
W303	*BDP1-3HA S164/S178A::kanR maf1Δ::natR* pRS313*MAF1-9myc*	this work	5E
W303	*BDP1-3HA S49/S586A::kanR maf1Δ::natR* pRS313*MAF1-9myc*	this work	5E
W303	*RPC160-3HA::hphR maf1Δ::kanR* pRS314*MAF1*	this work	5A, 5B
W303	*RPC160-3HA::hphR maf1Δ::kanR* pRS314*MAF1-9myc*	this work	5A, 5B

Table S1 continued

W303	*maf1Δ::kanR* pRS314*MAF1*	this work	5A, 5B
W303	*maf1Δ::kanR* pRS314*MAF1-9myc*	this work	5A, 5B
W303	*BDP1-3HA::kanR* pRS313	this work	S2A
W303	*BDP1-3HA::kanR 4SA* pRS313	this work	S2A
W303	*bdp1Δ::natR* pRS315*BDP1-3HA*	this work	S2B
W303	*bdp1Δ::natR* pRS315*BDP1-3HA 4SE*	this work	S2B
W303	*BDP1-3HA::kanR maf1Δ::natR* pRS313*MAF1-9myc*	this work	S2C
W303	*BDP1-3HA::kanR 4SE maf1Δ::natR* pRS313*MAF1-9myc*	this work	S2C
W303	*BDP1-3HA::kanR maf1Δ::natR* pRS313*MAF1-9myc 6SE*	this work	S2C
W303	*BDP1-3HA::kanR 4SE maf1Δ::natR* pRS313*MAF1-9myc 6SE*	this work	S2C
W303	*BDP1-3HA::kanR maf1Δ::natR* pRS313*MAF1-9myc 7SE*	this work	S2C
W303	*BDP1-3HA::kanR 4SE maf1Δ::natR* pRS313*MAF1-9myc 7SE*	this work	S2C

1 Lee, J., R. D. Moir, K. B. McIntosh and I. M. Willis (2012). "TOR signaling regulates ribosome and tRNA synthesis via LAMMER/Clk and GSK-3 family kinases." *Mol Cell* **45**(6): 836-843.

Desai, N., J. Lee, R. Upadhya, Y. Chu, R. D. Moir and I. M. Willis (2005). "Two steps in Maf1-dependent repression of transcription by RNA polymerase III." *J Biol Chem* **280**(8): 6455-6462.

Moir, R. D., J. H. Lee, R. A. Haeusler, N. Desai, D. R. Engelke and I. M. Willis (2006). "Protein kinase A regulates RNA polymerase III transcription through the nuclear localization of Maf1." *Proc. Natl. Acad. Sci. USA* **103**(41): 15044-15049.

Lee, J., R. D. Moir and I. M. Willis (2009). "Regulation of RNA polymerase III transcription involves SCH9-dependent and SCH9-independent branches of the target of rapamycin (TOR) pathway." *J Biol Chem* **284**(19): 12604-12608.

Moir, R. D., J. Lee and I. M. Willis (2012). "Recovery of RNA polymerase III transcription from the glycerol-repressed state: revisiting the role of protein kinase CK2 in Maf1 phosphoregulation." *J Biol Chem* **287**(36): 30833-30841.
