# Supplementary material for: Differential Phosphorylation of RNA Polymerase III and the Initiation Factor TFIIIB in Saccharomyces cerevisiae
Source: PLoS One. 2015 May 13;10(5):e0127225. doi: 10.1371/journal.pone.0127225 (PMC4430316; doi:10.1371/journal.pone.0127225)
Supplement: S2 Table — (PDF) [file pone.0127225.s006.pdf]

Table S2. Summary of Yeast RNA Polymerase III and TFIIIB Phosphorylation

Systematic name	Standard name	Gene product Alias	Mapped phosphosites[1] (amino acid residue)	Molecules per cell[2]	Doubling time (min)[3]	In vivo ^{32}P labeling[4]	Phos-tag bands
YOR116C	RPO31	C160	9, 1053	6020	97	nd	1
YOR207C	RET1	C128	229, 231, 235, 972	1950	96	nd	1
YPR190C	RPC82	C82	27, 106, 110, 392, 394, 399, 402, 430	2520	96	+	3
YDL150W	RPC53	C53	27, 116, 119, 130, 137, 138, 178, 181, 182, 224, 228, 232, 234, 347	998	98	+	2
YPR110C	RPC40	AC40	13, 15, 16, 17, 127	1300	88	+	2
YKR025C	RPC37	C37	18, 52, 61	1590	99	nd	1
YNR003C	RPC34	C34	nd	na	97	nd	1
YNL151C	RPC31	C31	101, 189, 190	na	151	+	1
YBR154C	RPB5	ABC27	nd	na	88	nd	1
YKL144C	RPC25	C25	162	752	91	nd	1
YPR187W	RPO26	ABC23	24, 102	na	181	+	1
YNL113W	RPC19	AC19	10, 15, 33, 51, 54, 55	8680	118	+	2
YJL011C	RPC17	C17	nd	2930	153	nd	1
YOR224C	RPB8	ABC14.5	68	6210	101	nd	2
YDR045C	RPC11	C11	nd	na	153	nd	2
YHR143W-A	RPC10	ABC10α	20, 35	5300	125	nd	1
YOR210W	RPB10	ABC10β	nd	1720	99	nd	1
YER148W	SPT15	TBP	42, 209	9000	92	na	1
YGR246C	BRF1	Brf1	375, 381, 384	13000	97	na	2
YNL039W	BDP1	Bdp1	33, 49, 73, 164, 178, 586	10000	111	na	4

nd, not detected; na, not available.

1. Data extracted from PhosphoGrid [1] and PhosphoPep [2] databases (accessed November 1, 2013) and published papers[3-6].
2. Data for RNA pol III [7] and for TFIIIB [8].
3. BY4741, 92 min. is the reference doubling time for all strains except for Brf1 and Bdp1 where W303, 104 min is the reference.
4. Data from [9,10].

1. Stark C, Su TC, Breitkreutz A, Lourenco P, Dahabieh M, et al. (2010) PhosphoGRID: a database of experimentally verified in vivo protein phosphorylation sites from the budding yeast Saccharomyces cerevisiae. Database (Oxford) 2010: bap026.
2. Bodenmiller B, Campbell D, Gerrits B, Lam H, Jovanovic M, et al. (2008) PhosphoPep--a database of protein phosphorylation sites in model organisms. Nat Biotechnol 26: 1339-1340.
3. Helbig AO, Rosati S, Pijnappel PW, van Breukelen B, Timmers MH, et al. (2010) Perturbation of the yeast N-acetyltransferase NatB induces elevation of protein phosphorylation levels. BMC Genomics 11: 685.
4. Holt LJ, Tuch BB, Villen J, Johnson AD, Gygi SP, et al. (2009) Global analysis of Cdk1 substrate phosphorylation sites provides insights into evolution. Science 325: 1682-1686.
5. Mohammed S, Lorenzen K, Kerkhoven R, van Breukelen B, Vannini A, et al. (2008) Multiplexed proteomics mapping of yeast RNA polymerase II and III allows near-complete sequence coverage and reveals several novel phosphorylation sites. Analytical Chemistry 80: 3584-3592.
6. Soufi B, Kelstrup CD, Stoehr G, Frohlich F, Walther TC, et al. (2009) Global analysis of the yeast osmotic stress response by quantitative proteomics. Mol Biosyst 5: 1337-1346.
7. Ghaemmaghami S, Huh WK, Bower K, Howson RW, Belle A, et al. (2003) Global analysis of protein expression in yeast. Nature 425: 737-741.
8. Sethy-Coraci I, Moir RD, Lopez-de-Leon A, Willis IM (1998) A differential response of wild type and mutant promoters to TFIIIB70 overexpression in vivo and in vitro. Nucleic Acids Res 26: 2344-2352.
9. Chedin S, Ferri ML, Peyroche G, Andrau JC, Jourdain S, et al. (1998) The yeast RNA polymerase III transcription machinery: a paradigm for eukaryotic gene activation. Cold Spring Harb Symp Quant Biol 63: 381-389.
10. Lee J, Moir RD, McIntosh KB, Willis IM (2012) TOR signaling regulates ribosome and tRNA synthesis via LAMMER/Clk and GSK-3 family kinases. Mol Cell 45: 836-843.
